# Effects of stress during pregnancy on hepatic glucogenic capacity in rat dams and their fetuses

**DOI:** 10.14814/phy2.13293

**Published:** 2017-06-14

**Authors:** Kathryn L. Franko, Alison J. Forhead, Abigail L. Fowden

**Affiliations:** ^1^ Department of PhysiologyDevelopment and NeuroscienceUniversity of CambridgeCambridgeUnited Kingdom

**Keywords:** Glucogenic capacity, Pregnancy, Stress

## Abstract

Stress during pregnancy is associated with metabolic dysfunction in the adult offspring in human and other animals. However, little is known about the metabolic effects of pregnancy stress on the mothers and fetuses during pregnancy itself. This study aimed to determine the consequences of the common experimental procedures of injection and single housing in pregnant rats on fetal and maternal hepatic glucogenic capacities. On day (D) 20 of pregnancy, feto‐placental weights and the glycogen content and activities of phosphoenolpyruvate carboxykinase (PEPCK) and glucose‐6‐phosphatase (G6Pase) of fetal and maternal liver were measured in rats pair or single housed from D1 with or without saline injection from D15 to D19. Housing and saline injection both affected hepatic glucogenic capacity. In maternal liver, saline injection but not housing reduced glycogen content and raised G6Pase activity, whereas housing but not treatment increased PEPCK activity. In fetuses, housing and injection interacted in regulating PEPCK activity and reducing hepatic glycogen content and placental weight. Body weight was decreased and hepatic G6Pase increased by injection but not housing in the fetuses. Single‐housed dams ate less than those pair‐housed near term while saline injection elevated maternal plasma corticosterone concentrations. Thus, single housing and saline injection are both stresses during rat pregnancy that alter feto‐placental weight and hepatic glucogenic capacity of the fetuses and dams near term. Routine experimental procedures per se may, therefore, have consequences for offspring hepatic phenotype as well as modifying the outcomes of dietary and other environmental challenges during pregnancy.

## Introduction

Human epidemiological studies have shown that stress during pregnancy is often associated with low birth weight and an increased risk of adult‐onset and other diseases, such as glucose intolerance, Type 2 diabetes, asthma, hypertension, and behavioral disorders (Barker 1994; Martin‐Gronert and Ozanne 2012; Hanson and Gluckman 2014; Mina and Reynolds 2014; Vaiserman 2015; Andersson et al. 2016). In experimental animals, intrauterine programming of adult glucoregulatory mechanisms has been demonstrated in a number of species using a range of techniques to induce maternal stress during pregnancy (Reusens and Remacle 2001; McMillen and Robinson 2005; Martin‐Gronert and Ozanne 2012; Mina and Reynolds 2014). In rats, maternal restraint, dietary manipulation, and direct glucocorticoid administration have all been shown to cause postnatal abnormalities in hepatic glucose metabolism in the offspring consistent with the human epidemiological data (McMillen and Robinson 2005; Martin‐Gronert and Ozanne 2012; Mina and Reynolds 2014). In particular, rats exposed in utero to these stresses are glucose intolerant with increased hepatic gluconeogenic enzyme activities and abnormal responses to glucoregulatory hormones as adults (Burns et al. 1997; Desai et al. 1997; Nyirenda et al. 1998; Lesage et al. 2004; D'Mello and Liu 2006; Franko et al. 2010). Increased hepatic gluconeogenic enzyme activities are also seen in rat fetuses when growth is restricted by maternal protein deprivation, indicating that the postnatal abnormalities may arise in utero (Franko et al. 2009).

Experimental procedures, such as individual housing, handling, and saline injection, frequently used as controls in developmental studies, can be stressful and cause changes in food intake and raised corticosterone concentrations in pregnant and nonpregnant rats (Ward and Weisz 1984; Barbazanges et al. 1996). Glucocorticoids and undernutrition are known to alter hepatic glycogen content and enhance gluconeogeneic enzyme activities in nonpregnant adult animals (Weber and Cantero 1957; Perez et al. 1964; Oh et al. 1970; Franko et al. 2007). However, the extent to which routine experimental procedures cause stress during pregnancy and impairs fetal‐placental development and enhances hepatic glucogenic capacity of the mother and fetus remains largely unknown. This study, therefore, tested the hypothesis that two common experimental procedures, single housing and saline injection, would alter feto‐placental growth and hepatic glucogenic capacity of rat dams and their fetuses near term. Hepatic glycogen content and activities of the key rate‐limiting glucogenic enzymes, phophoenolpyruvate caboxykinase (PEPCK) and glucose‐6‐phosphate (G6Pase), were measured in fetal and maternal liver from rats housed individually with or without saline injection near term and compared to similarly treated dams more naturally housed in pairs.

## Materials and Methods

### Animals

Twenty‐nine virgin female Wistar rats aged 12–15 weeks were used (Charles Rivers, Saffron Walden UK). They were maintained at 22°C on a 12 h light/dark cycle and fed a standard laboratory chow throughout (Standard Breeding Diet No 3, Special Diet Services, Essex, UK). Females were mated overnight with a male Wistar rat. The presence of a copulatory plug was taken as day (D) 0 of pregnancy (term is D21.5). All dams were weighed on D0 and D20. Food intake was measured for each cage and calculated as gram per rat per day. All studies were carried out under the Animal (Scientific Procedures) Act 1986 of the UK Government after ethical approval of the Animal Welfare and Ethical Review Board of the University of Cambridge.

### Experimental procedures

Before mating, all females were group housed, whereas, afterward, they were housed either individually (*n* = 13) or in pairs (*n* = 16 rats; eight pairs) in identical cages. Between 08.00 and 09.00 h on D15 to D19 inclusive, seven single‐housed and eight pair‐housed dams (both animals from four cages) were injected subcutaneously with saline (0.9% w/v NaCl, 200 *μ*L/100 g body weight). Between 08.00 and 10.00 h on D20, all dams were anesthetized with a mixture of sodium pentobarbitone and chloral hydrate (Equithesin, 0.6 mL/100 g body weight, ip). Once anesthetized, a 2 mL blood sample was collected from the uterine vein for measurement of blood glucose and plasma corticosterone concentrations. Fetuses and placentas were removed individually and weighed. Fetuses were killed by decapitation and their glucose concentration measured in blood from the severed neck vessels. The fetal liver was removed, weighed, and frozen in liquid nitrogen for subsequent analyses of glycogen content and gluconeogenic enzyme activities. The fetuses were sexed visually after removal of the liver. After delivery of the entire litter, the dam was killed with a lethal dose of anesthetic (200 mg/kg sodium pentobarbitone, Dolthetal, Ventoquinol, UK Ltd). Maternal liver was collected and frozen in liquid nitrogen. Maternal blood samples were centrifuged at 4°C and the plasma stored at −20°C. One of the pair‐housed, saline injected dams had an abnormally small litter (five pups) so this animal was excluded from any further analyses. Food intake for this cage was also discounted. The numbers of dams in each group was, therefore, as follows: pair housed and untreated (UT) *n* = 8 dams, pair housed and saline injected (INJ) *n* = 7 dams, single housed and untreated *n* = 6 dams, and, finally, single housed and saline injected *n* = 7 dams.

### Biochemical analyses

Blood glucose concentrations were measured using a hand‐held glucometer (Lifespan, Ortho‐Clinical Diagnostics, High Wycombe, UK). The hepatic glycogen content and activities of G6Pase (EC 3.1.3.9) and PEPCK (EC 4.1.1.49) were measured in all dams and one male and one female fetus per litter per variable (chosen randomly) using established methods described in detail previously (Fowden et al. 1993; Franko et al. 2007, 2009). The interassay coefficients of variation of a fetal homogenate in the glycogen, G6Pase, and PEPCK assays were 9%, 11.8%, and 5%, respectively. Hepatic protein content was determined using a Lowry assay. Plasma corticosterone concentrations were measured by radioimmunoassay using a single kit (ImmuChem, Orangeburg, NY). The lower limit of sensitivity of the assay was 10 ng/mL and the intraassay coefficient of variation was <10%. All biochemical and hormone analyses were made in duplicate.

### Statistical analyses

Mean (±SE) values have been used throughout. Maternal data were analyzed by two‐way ANOVA with housing and treatment as factors using the Holm–Sidak post hoc test for all subsequent pair‐wise comparisons. Initially, fetal data were analyzed by three‐way ANOVA with housing, treatment, and sex of the fetus as factors. This showed no effect of sex, or any interaction between sex and the other two factors, in determining any of morphometric or biochemical variables measured. Consequently, for fetal data, statistical analyses used litter means irrespective of sex for comparisons between groups using two‐way ANOVA as for the maternal data. For fetal morphometric and glucose data, the whole litter was averaged while, for the hepatic variables, values for the two fetuses assayed per litter were averaged. Correlation between variables was assessed by Pearson's correlation analyses. Statistical significance was accepted when *P* < 0.05 (Sigma Stat 3.5, Systat Software, Point Richmond, CA).

## Results

### Morphometry and biochemical composition

Maternal body weight was similar in all four groups on D0 (Table 1). However, by D20, maternal body weight was greater in dams housed in pairs than singly (Table 1). Maternal weight gain between D0 and D20 was influenced by both housing and injection with smaller weight gains in dams housed singly than in pairs and in those saline‐injected relative to untreated dams (Table 1). Maternal food intake was similar in all four groups until D15 but, thereafter, singly housed dams ate less than those pair housed, irrespective of whether the animals were injected or not (Table 1). Litter size did not differ between groups (Table 1). Housing and injection interacted in determining maternal hepatic protein content with significantly lower values in singly housed, saline‐injected dams than in their untreated or pair‐housed counterparts (Table 1). Maternal corticosterone concentrations were significantly higher in saline‐injected than uninjected dams, irrespective of housing (Table 1). There were no significant differences in maternal or fetal blood glucose concentrations between groups (Table 1 & 2).

**Table 1:** The effect of stress during pregnancy on maternal characteristics

	Pair housed		Single housed		Significance		
	Uninjected	Injected	Uninjected	Injected	Housing	Injection	Interaction
Body weight g							
Day 0	277 ± 14	286 ± 9	271 ± 16	266 ± 15	NS	NS	NS
Day 20	428 ± 20	405 ± 23	398 ± 18	366 ± 22	**0.011**	NS	NS
Gain 0–20	150 ± 13	125 ± 8	128 ± 11	100 ± 17	**0.034**	**0.019**	NS
Food intake g/day/rat							
0–10 days	23.8 ± 0.8	23.3 ± 1.3	21.5 ± 0.8	21.1 ± 0.7	NS	NS	NS
11–15 days	26.4 ± 2.0	27.8 ± 2.2	24.8 ± 1.6	23.5 ± 1.2	NS	NS	NS
16–20 days	30.7 ± 1.9	30.8 ± 2.6	25.6 ± 1.0	24.4 ± 1.6	**0.007**	NS	NS
Litter size	14.4 ± 1.0	14.6 ± 0.8	14.3 ± 0.8	14.0 ± 0.6	NS	NS	NS
Corticosterone ng/mL	571 ± 53	691 ± 55	565 ± 94	818 ± 51	NS	**0.016**	NS
Glucose mmol/L	4.18 ± 0.25	5.93 ± 0.88	4.90 ± 0.63	5.38 ± 1.27	NS	NS	NS
Liver							
Protein content mg/g	162.1 ± 5.5	171.6 ± 5.2	168.4 ± 12.5	145.3 ± 5.9^a^, ^b^	NS	NS	**0.028**
G6Pase U/g wet wt	6.01 ± 0.51	9.71 ± 1.07	6.28 ± 1.32	9.22 ± 0.38	NS	**0.001**	NS
PEPCK U/g wet wt	0.96 ± 0.18	1.02 ± 0.17	2.72 ± 0.43	2.63 ± 0.37	**<0.001**	NS	NS

Mean (±SEM) values of body weight, weight gain, food intake, litter size, concentrations of plasma corticosterone and blood glucose and of hepatic protein content, and activities of glucose‐6‐phosphatase (G6Pase) and phosphoenolpyruvate carboxykinase (PEPCK) per gram liver at day (D) 20 of pregnancy in rats that were pair or single housed from conception and either uninjected or injected with saline from D15 to D19. Pair housed: untreated *n* = 8, saline injected *n* = 7 for dams and untreated *n* = 4, saline injected *n* = 3 for cages for food intake; Single housed: untreated *n* = 6, saline injected *n* = 7 for dams and cages. Statistical significance was assessed by two‐way ANOVA with Holm–Sidak post hoc test. NS = Not significant. Significant *P* values for effects are shown in bold.

a Interactions: Significantly different from the value in the uninjected group in the same housing conditions (*P* < 0.01).

b Interactions: Significantly different from the value in saline‐injected pair housed groups (*P* < 0.01).

**Table 2:** The effects of stress during pregnancy on fetal characteristics

	Pair housed		Single housed		Significance		
	Uninjected	Injected	Uninjected	Injected	Housing	Injection	Interaction
Placental weight mg	733 ± 37	578 ± 25^a^	606 ± 47^b^	584 ± 11	NS	**0.008**	**0.038**
Body weight (BW) g	4.04 ± 0.18	3.48 ± 0.07	3.77 ± 0.08	3.60 ± 0.07	NS	**0.007**	NS
Liver weight							
mg	346 ± 15	290 ± 9	280 ± 13	276 ± 11	**0.017**	NS	NS
% BW	8.5 ± 0.2	8.3 ± 0.2	7.5 ± 0.2	7.7 ± 0.4	**0.002**	NS	NS
Liver protein content mg/g	101.8 ± 3.5	96.5 ± 6.7	112.9 ± 4.5	103.1 ± 2.2	NS	NS	NS
Liver Glycogen							
Total mg	30.4 ± 4.8	11.2 ± 1.2^a^	13.6 ± 1.4^b^	14.7 ± 2.0	**0.033**	**0.005**	**<0.001**
mg/g fetus	6.9 ± 0.7	3.2 ± 0.3^a^	3.7 ± 0.3^b^	4.1 ± 0.5	**0.036**	**0.007**	**0.001**
Liver G6Pase							
Total mU	408 ± 37	476 ± 103	389 ± 65	528 ± 35	NS	NS	NS
U/g liver	1.24 ± 0.10	1.55 ± 0.15	1.40 ± 0.25	1.89 ± 0.10	NS	**0.012**	NS
mU/g fetus	107 ± 8	136 ± 26	104 ± 18	146 ± 9	NS	**0.032**	NS
Liver PEPCK							
Total mU	95 ± 8	89 ± 15	51 ± 7^b^	124 ± 11^a^, ^b^	NS	**0.006**	**0.002**
U/g liver	0.30 ± 0.02	0.24 ± 0.04	0.18 ± 0.02^b^	0.46 ± 0.03^a^, ^b^	NS	**0.003**	**<0.001**
mU/g fetus	25 ± 2	25 ± 5	14 ± 2^b^	34 ± 3^a^, ^b^	NS	**0.001**	**0.002**
Glucose mmol/L	4.23 ± 0.61	3.78 ± 0.37	4.55 ± 0.18	4.20 ± 0.49	NS	NS	NS

Mean (±SEM) values of placental, fetal body and liver weights, blood glucose concentrations, and of hepatic glycogen content and activities of glucose‐6‐phosphatase (G6Pase) and phosphoenolpyruvate carboxykinase (PEPCK) expressed on an absolute and weight‐specific basis in rat fetuses at day 20 of gestation from dams that were pair or single housed from conception and either uninjected or injected with saline from days 15 to 19 of pregnancy. Pair housed: untreated *n* = 6–8 litters, saline injected *n* = 6–7 litters; Single housed: untreated *n* = 5–6 litters, saline injected *n* = 7 litters. Statistical significance was assessed by two‐way ANOVA with Holm–Sidak post hoc test. NS = Not significant. Significant *P* values of effects are shown in bold.

a Interactions: Significantly different from the value in the uninjected group in the same housing conditions (*P* < 0.01).

b Interactions: Significantly different from the value in the corresponding injected or uninjected pair housed group (*P* < 0.01).

On D20, placental weight was influenced significantly by injection and by interactions between injection and housing (Table 2). Placental weight was higher in uninjected than saline‐injected dams due primarily to an effect in pair‐housed animals (Table 2). Placental weight in uninjected dams was lower in singly than pair‐housed dams (Table 2). Fetal weight was also affected by injection with lower weights in injected than uninjected dams (Table 2). There was no significant interaction between housing and injection in determining fetal weight (Table 2). Fetal liver weight was influenced significantly by housing but not by treatment, whether expressed as absolute weight or as a percentage of body weight (Table 2). Fetal hepatic protein content did not differ between groups (Table 2).

### Glucogenic capacity

**Figure 1:**
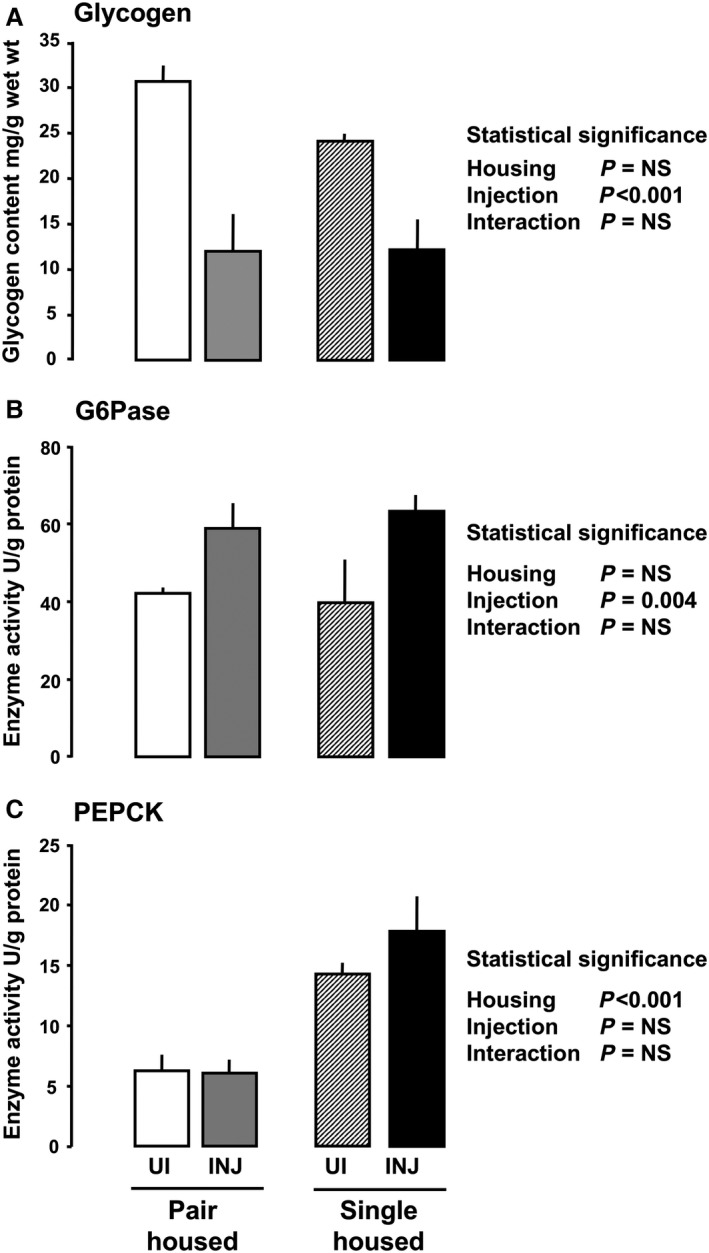
Mean (±SEM) maternal hepatic glycogen content (A) and activity of glucose‐6‐phosphatase (G6Pase, B) and phosphoenolpyruvate carboxykinase (PEPCK, C) at day (D) 20 of pregnancy with the statistical significance of the effects of housing, injection, and their interaction in rats housed singly or in pairs either uninjected (UI) or injected with saline (INJ) from D15 to D19 by two‐way ANOVA. NS = Not significant. (Pair housed: uninjected *n* = 8 dams, saline injected *n* = 7 dams; Singly housed: uninjected *n* = 6 dams, saline injected *n* = 7 dams).

**Figure 2:**
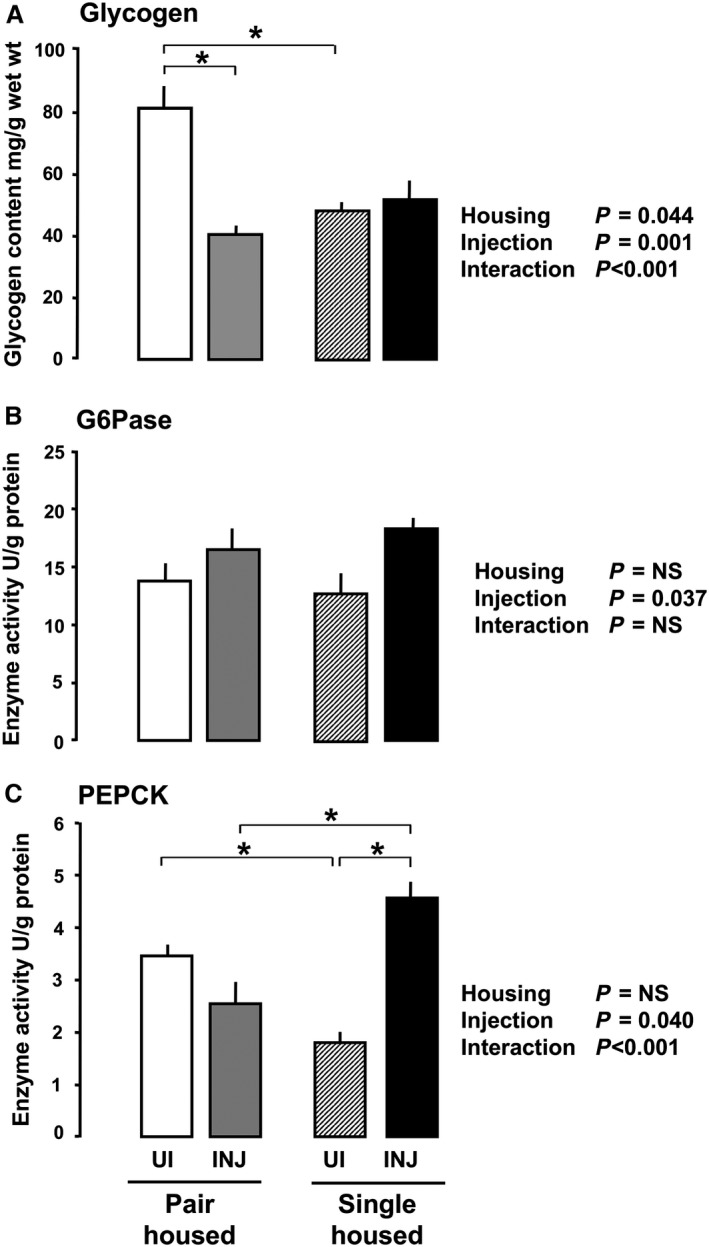
Mean (±SEM) fetal hepatic glycogen content (A) and activities of glucose‐6‐phosphatase (G6Pase, B) and phosphoenolpyruvate carboxykinase (PEPCK, C) activity at day (D) 20 of gestation with the statistical significance of the effects of housing, injection, and their interaction in rats housed singly or in pairs either uninjected (UI) or injected with saline (INJ) from D15 to D19 by two‐way ANOVA with Holm–Sidak post hoc test where *= *P* < 0.01 between‐individual groups when there was a significant interaction. NS = Not significant. (Pair‐housed: uninjected *n* = 7–8 litters, saline injected *n* = 6–7 litters; Singly housed: uninjected *n* = 5–6 litters, saline injected *n* = 7 litters).

At D20, maternal hepatic glycogen content per gram wet weight was significantly lower in saline‐injected than uninjected dams, irrespective of housing (Fig. 1A). Housing had no significant effect on maternal hepatic glycogen content and there was no interaction between housing and injection (Fig. 1A). In the fetuses, both housing and injection were significant influences on hepatic glycogen content whether expressed per gram wet weight of liver, per gram wet weight of fetus, or as total content (Fig. 2A, Table 2). There was also a significant interaction between these two factors in determining fetal hepatic glycogen content, irrespective of how values were expressed (Fig. 2A). Glycogen content per gram fetal liver was lower in injected than uninjected dams that were pair housed but not in those singly housed (Fig. 2A, Table 2). It was also lower in fetuses of untreated dams singly housed relative to their pair‐housed counterparts (Fig. 2A, Table 2).

Maternal hepatic G6Pase activity, whether expressed per gram protein or per gram wet weight of liver, was influenced significantly by injection with higher activities in saline‐injected than untreated dams, irrespective of housing (Fig. 1B, Table 1). In contrast, maternal hepatic PEPCK activity was unaffected by injection but was influenced by housing with significantly higher activities in singly than pair‐housed dams (Figure 1C, Table 1). There was no significant interaction between injection and housing in determining maternal hepatic activity of G6Pase or PEPCK (Fig. 1B & C, Table 1). Maternal hepatic activity of G6Pase, but not PEPCK, was positively correlated with maternal plasma corticosterone concentration, irrespective of whether activity was expressed per gram protein (G6Pase, *r* = 0.686, *P* < 0.01, *n* = 24; PEPCK, *r* = 0.443, *P* > 0.05, *n* = 23) or per gram wet weight of liver (G6Pase, *r* = 0.614, *P* < 0.01, *n* = 24; PEPCK, *r* = 0.401, *P* > 0.05, *n* = 23). There were no correlations between maternal blood glucose concentrations and hepatic activities of G6Pase or PEPCK (*P* > 0.05, all cases).

Like the dams, fetal hepatic G6Pase expressed either per gram protein or per gram wet weight of liver was higher in saline‐injected than uninjected groups and was unaffected by housing (Fig. 2B, Table 2). In contrast, fetal hepatic PEPCK activity per gram protein or per gram wet weight of liver was affected significantly by injection in a manner that depended on housing (Fig. 2C, Table 2). In injected groups, fetal hepatic PEPCK activity was higher in singly than pair‐housed dams, whereas, the reverse was seen in uninjected groups (Fig. 2C, Table 2). There were no correlations between fetal blood glucose concentrations and activity of either enzyme however values were expressed (*P* > 0.05, all cases).

## Discussion

The results demonstrate that both single housing and saline injection of pregnant rats alter the hepatic glucogenic capacity of maternal and fetal liver near term. There were also interactions between housing and injection in determining hepatic activities of G6Pase and PEPCK. These changes were accompanied by alterations in feto‐placental growth, which may have contributed to the fetal hepatic phenotype. Compared to uninjected pair‐housed dams, single housing and saline injection both reduced placental growth. However, fetal weight was reduced significantly only in the injected dams and not in the single‐housed uninjected dams that also had reduced placental weight. The changes in hepatic glucogenic capacity were also accompanied by reduced food intake in late gestation during single housing and by increased corticosterone concentrations in saline‐injected dams. These findings indicate that, single housing and saline injection are both stressors in pregnant rats, which alter growth and hepatic metabolism of the mother and her fetuses with potential consequences for their glucose metabolism after birth.

In maternal liver, saline injection reduced glycogen content and increased G6Pase activity, irrespective of housing conditions. Given the short time frame between anesthesia and tissue collection and the finding that maternal glucose concentrations immediately after anesthesia were unaffected by daily injection, the low hepatic glycogen levels of the saline‐injected dams are unlikely to be due to hepatic glycogenolysis during the acute terminal procedures. They are more likely to reflect accumulated effects of enhanced glycogenolysis and/or decreased glycogen synthesis in response to the daily stress of handling and saline injection over the preceding 5 days, particularly as corticosterone concentrations were raised in these dams. These higher corticosterone concentrations may also explain the elevated hepatic G6Pase activity of injected dams as glucocorticoids stimulate hepatic G6Pase activity in nonpregnant rats (Weber and Cantero 1957). Certainly, there was a significant positive correlation between corticosterone concentrations and hepatic G6Pase activity in the pregnant dams used here. In contrast, maternal hepatic PEPCK activity was unaffected by injection but was two‐ to threefold higher in singly than pair‐housed groups. These changes may reflect maternal nutritional state as food intake was lower in singly housed dams during late gestation. Previous studies in nonpregnant rats have shown that reducing dietary carbohydrate intake increases hepatic PEPCK activity (Perez et al. 1964; Lanoue et al. 1999). Collectively, the current findings indicate that both corticosterone and lower nutrient intake are important influences on maternal glucogenic capacity during rat pregnancy, but that they act differentially on the two enzymes studied with hepatic G6Pase, the final rate‐limiting enzyme of both glycogenolysis and gluconeogenesis, more sensitive to glucocorticoids and PEPCK, the rate‐limiting enzyme specifically of gluconeogenesis, more responsive to undernutrition. This is consistent with the known roles of glycogenolysis as an immediate source of extra glucose during stress and gluconeogenesis as a longer‐term source of glucose for essential processes when its dietary availability is restricted (Perez et al. 1964).

Single housing and injection of the dams also impaired feto‐placental growth in line with the reduced maternal weight gain during pregnancy in these groups. Again, these effects on conceptus growth were probably related to the reduced maternal food intake and raised corticosterone concentrations. Both these factors are known to reduce placental and fetal weight in rats (Nyirenda et al. 1998; Ain et al. 2005; Belkacemi et al. 2012). Recent studies in pregnant mice have also shown that, while maternal undernutrition and corticosterone administration produce a similar reduction in placental weight, their effects on placental nutrient transfer capacity differ (Coan et al. 2010; Vaughan et al. 2012). Corticosterone administration reduced placental amino acid transport, whereas undernutrition increased amino acid transfer across the small placenta, which helped to sustain fetal growth in the undernourished animals (Coan et al. 2010; Vaughan et al. 2012). These observations are consistent with the current findings that fetal weight was only influenced by injection with no interaction with housing. Thus, fetal growth may have been maintained in uninjected single‐housed dams with reduced food intake by upregulating amino acid transfer across their small placenta. However, the alterations in fetal growth may not be due solely to placental changes. The raised maternal corticosterone concentrations may also have a more direct inhibitory effect on fetal growth as transplacental corticosterone transfer occurs in pregnant rats during restraint stress and prolonged dietary restriction (Ward and Weisz 1984; Lesage et al. 2001; Straud et al. 2006; Belkacemi et al. 2012). Whatever the cause, impaired placental growth and function would decrease total substrate availability for fetal growth and deposition of fuel reserves, such as glycogen (Oh et al. 1970). Indeed, in this study, fetal hepatic glycogen content was low in all groups with reduced placental weight.

Fetal hepatic G6Pase mirrored maternal values with higher activities in saline‐injected groups irrespective of housing. In contrast, fetal hepatic PEPCK activity differed in profile from the maternal activities with injection interacting with housing in the fetuses. This suggests that, unlike the dams, both an impaired nutrient supply and increased corticosterone exposure are required to increase hepatic PEPCK activity in the fetus, a scenario which would preserve amino acids for fetal tissue accretion for as long as possible. Nutrition and glucocorticoids are known to regulate fetal glucogenic capacity in species more mature than rodents at birth (Fowden et al. 1998). In fetal sheep and pigs, hepatic G6Pase activity and glycogen deposition are glucocorticoid dependent and are increased by fetal and maternal glucocorticoid treatment in late gestation (Fowden et al. 1993, 1995; Franko et al. 2010). Fetal hepatic PEPCK activity is also increased by cortisol overexposure in these species, although it appears to be less glucocorticoid sensitive than G6Pase activity (Fowden et al. 1993; Franko et al. 2010). Hepatic PEPCK gene expression is also increased in term rat pups by preimplantation dietary protein deprivation (Kwong et al. 2007). Collectively, the current findings suggest that fetal hepatic G6Pase and PEPCK activities may have similar nutritional and glucocorticoid sensitivities in rats as seen in other species, as the highest activities were seen in fetuses exposed to lower maternal food intake and greater maternal corticosterone concentrations. Indeed, upregulation of these hepatic enzyme activities in fetuses of the singly housed, saline‐injected rat dams more than compensated for the reduced fetal liver weight as enzyme activity per gram fetus was highest in this group. This may also offset, in part, the limited glycogen availability for hepatic glucogenesis. If these adaptations persist after birth, they may have consequences for postnatal glucose metabolism similar to those seen in adult rats which were growth retarded in utero by more severe nutritional and glucocorticoid treatments (Desai et al. 1997; Nyirenda et al. 1998; Gallo et al. 2012).

In summary, the current findings show that the experimental procedures commonly used as controls in studying developmental programming are stressful and will themselves alter fetal hepatic development. Most rodent studies of developmental programming are, therefore, examining the effects of adverse environmental superimposed upon unnatural conditions created by the experimental procedures per se. This combination of pregnancy stresses may result in a different metabolic phenotype than either stress alone and explain, in part, the variations in offspring outcomes seen after apparently similar maternal dietary or other manipulations during pregnancy (McMillen and Robinson 2005; Mina and Reynolds 2014). For example, in this study, the effect of maternal saline injection on the hepatic glucogenic capacity of the fetuses depended on the housing conditions. This study also demonstrates that developmental programming by suboptimal conditions may occur directly via changes in the fetus or indirectly through altered maternal metabolism or placental development. It also emphasizes sensitivity of hepatic development to environmental cues unnatural in an evolutionary context in rodents and highlights the potential role of experimental procedures per se in modifying the phenotypical outcome of environmental challenges during pregnancy.

## Conflict of Interest

None declared.
